# Supramolecular organization of NMDA receptors and the postsynaptic density

**DOI:** 10.1016/j.conb.2017.05.019

**Published:** 2017-05-31

**Authors:** René AW Frank, Seth GN Grant

**Affiliations:** 1 MRC Laboratory of Molecular Biology, Francis Crick Avenue, Cambridge CB2 0QH, UK; 2 Centre for Clinical Brain Sciences, University of Edinburgh, Chancellor’s Building, Edinburgh EH16 4SB, UK

## Abstract

The postsynaptic density (PSD) of all vertebrate species share a highly complex proteome with ~1000 conserved proteins that function as sophisticated molecular computational devices. Here, we review recent studies showing that this complexity can be understood in terms of the supramolecular organization of proteins, which self-assemble within a hierarchy of different length scales, including complexes, supercomplexes and nanodomains. We highlight how genetic and biochemical approaches in mice are being used to uncover the native molecular architecture of the synapse, revealing hitherto unknown molecular structures, including highly selective mechanisms for specifying the assembly of NMDAR-MAGUK supercomplexes. We propose there exists a logical framework that precisely dictates the subunit composition of synaptic complexes, supercomplexes, and nanodomains *in vivo*.

## Introduction

In the early 1990s the available evidence suggested that a handful of postsynaptic proteins were sufficient for the functions of synaptic transmission and plasticity at excitatory synapses in the brain. Fast synaptic transmission mediated by AMPA subtypes of ionotropic glutamate receptors could be modulated by Ca^2+^-calmodulin Kinase II (CamKII) that was triggered by Ca^2+^ influx via the *N*-methyl-d-aspartic acid receptor (NMDAR) [1,2]. Glutamate receptors and the CamKII holoenzyme were each recognised to be multiprotein complexes comprised of receptor and kinase subunits respectively. However, the discovery that the NMDAR physically associates with many dozens of proteins [3,4,5••] led to the realization that receptors associated with vast numbers of different proteins, which included other complexes such as ion channels, adhesion and signalling proteins [3]. Furthermore, the vertebrate postsynaptic proteome was found to be far more complex than anticipated and is comprised of ~1000 highly conserved proteins in mice [6–8], rats [9,10], humans [7,11] and zebrafish [12]. It is highly unlikely that this molecular machinery simply support a generic function of transmission, because postsynaptic protein mutations in mice result in differential functional and behavioural phenotypes [13]. Moreover, at least 130 brain diseases, including common and rare psychiatric and neurological conditions, present with cognitive, motor, emotional phenotypes [11]. Thus, understanding the organization of the postsynaptic proteome is of fundamental importance to disease and the molecular basis of cognitive function.

**Figure 1:**
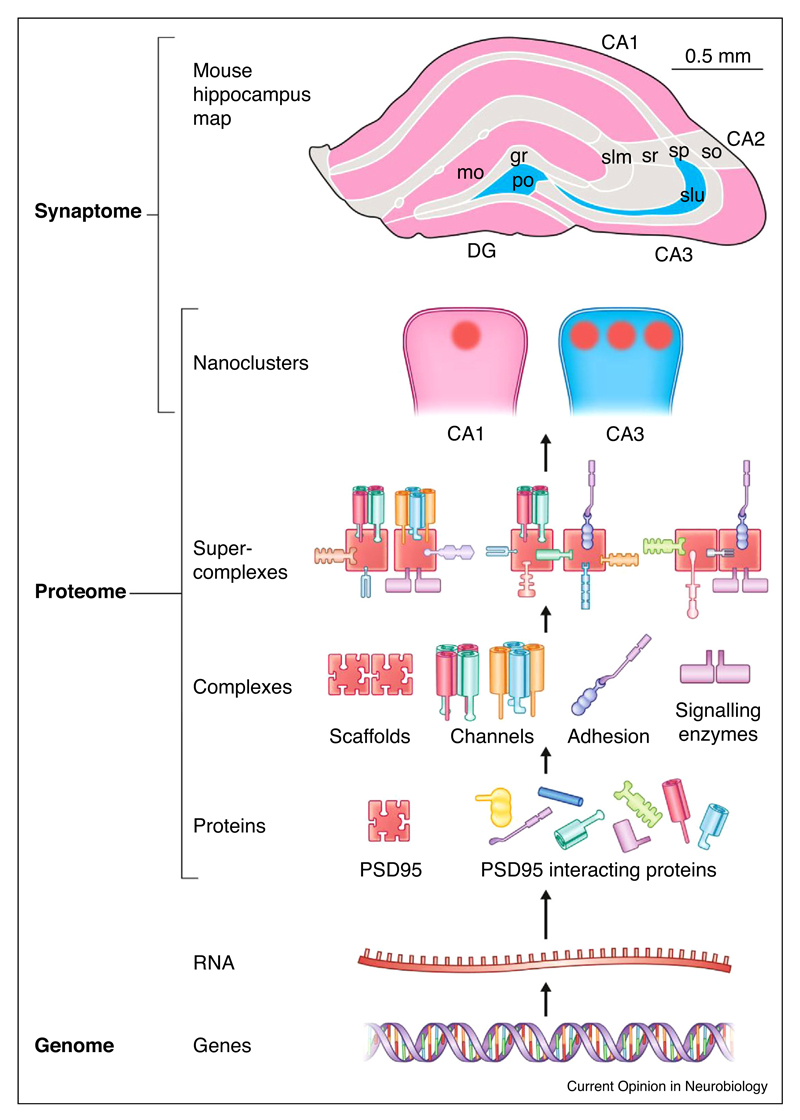
Hierarchy of supramolecular organization in the postsynaptic proteome. The genome and transcriptome encode individual proteins and instructs their hierarchical organization at different length scales into complexes, supercomplexes and nanoclusters. Different synapses express different numbers of nanoclusters and these synapses are differentially distributed into different brain regions, as indicated by the colour scheme (pink, regions of brain with predominantly single nanocluster synapses; blue, regions with multiple nanoclusters) of the mouse hippocampus (adapted from Ref. [44•]).

How are the vast numbers of postsynaptic proteins physically organised in the postsynaptic terminal? Do they constitute a ‘soup’ of different proteins or is there a molecular logic to the way they interact and function? Over the last thirty years it has become apparent that individual proteins are rarely deployed alone, but instead execute their functions within complexes and other higher-order molecular machines [14,15]. To generate a molecular machine, individual protein subunits assemble into complexes and these in turn associate to form supercomplexes (complexes of complexes) (Figure 1). Supercomplexes can be mega-Daltons in mass and perform fundamental biological processes, as exemplified by the respirasome, nuclear pore, proteasome, ribosome and spliceosome [14]. In contrast to these supercomplexes that are readily studied using cultured eukaryotic cells, the supercomplexes of the synapse have been particularly challenging to study because of the inherent complexity of brain tissue and the low abundance of the supercomplexes.

## Studying the supramolecular organisation of postsynaptic proteins

**Figure 2:**
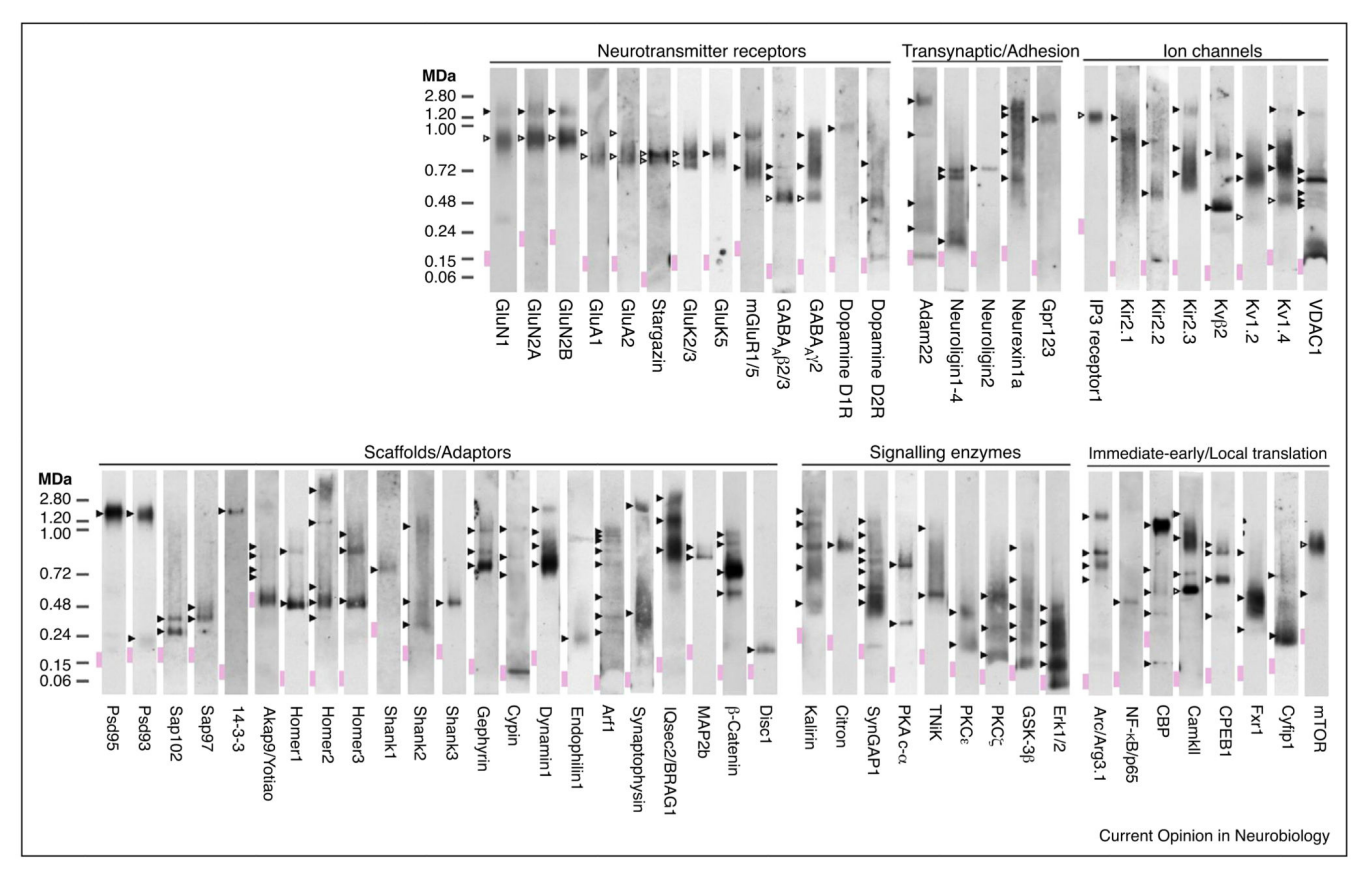
Supramolecular ‘fingerprint’ of 65 forebrain proteins. Adapted with permission from Ref. [5]. Native assemblies were detected by blue non-native PAGE immunoblot of mouse forebrain extracted with various different detergents. Expected and unexpected/unknown native protein assemblies within each lane are indicated by open and filled arrowheads, respectively. Native molecular mass indicated in mega-Daltons (MDa).

The majority of studies examining protein interactions and protein assemblies in the synapse have relied on *in vitro* methods, including yeast-2-hybrid interaction and pull-down assays. While useful in identifying potential binary interactions, these methods often mislead or do not accurately reflect the organization of proteins *in vivo*, especially when interactions are multivalent and involve more than two components acting in concert. An important insight into how the vast number of postsynaptic proteins are physically organized was obtained by a biochemical screen using Blue Native PAGE (BNP) to catalogue many functional classes including neurotransmitter receptors, trans-synaptic/adhesion, ion channels, signaling enzymes, scaffolds/adaptors and immediate-early/local translation proteins [5••]. Strikingly, 220 mouse forebrain synaptic complexes and supercomplexes (5–20x the monomer size) were evident of which only seventeen were previously known (Figure 2). These data provide a molecular blueprint for further interrogation of the assembly of the synapse. For example, this screen showed that GABA_A_ receptor subunits found at inhibitory synapses partition between ~500 kDa and ~720 and ~900 kDa native complexes [5••]. A more recent report elegantly analyzed the composition of the ~720 kDa native GABA_A_ receptor complex identifying neuroligin-2 and putative auxiliary GABA_A_ receptor subunits [16]. Thus, supramolecular assembly is likely to be a general property of synaptic proteins.

The major challenge is to biochemically isolate, identify and characterise the constituents of these myriad complexes and supercomplexes. Mass spectrometry of immunoprecipitated complexes, used to identify constituents of these novel complexes [3] is critically dependent on the efficiency and specificity of the antibody and the availability or viability of knockout mice that are needed in most cases to serve as negative controls. Nonetheless, this approach has been particularly successful at identifying auxiliary subunits of receptors found in various sub-compartments of the neuron, including AMPA receptors [17–19,20••], kainic acid receptors [21], GABA_B_ receptors [22], GABA_A_ receptors [16], and BK-Ca_v_ channel-channel supercomplexes [23]. Except in rare cases where peptide epitopes enable native elution [16,20••,^24^], a disadvantage of this approach is that samples must be denatured, which breaks apart the native complexes. This limiting factor can prevent further study of the biochemical and physiological function of complexes, as well as the identification of further subfamilies within a population of complexes.

A method that can overcome these limitations is to genetically modify the protein of interest so that it is fused with a protein sequence that is well suited to affinity purification and elution. Originally devised for use in *in vitro* systems, these protein sequences have been engineered into the genome of mice using gene-targeting methods, thereby ‘tagging’ endogenous proteins and their native assemblies. Typically, these tags are small domains encoding high-affinity binding sites and when used in tandem enable multiple steps of purification [25]. For example, a commonly used tandem-affinity purification (TAP)-tag includes Flag epitopes and hexahistidine tags, which can be used for antibody and nickel affinity purification, respectively. In this setting, wild type mice serve as excellent negative controls for purifying endogenous complexes. A key advantage in the design of these gene-tags is the use of epitopes including Flag, for which there are peptide reagents that trigger elution by competing for binding to antibody-coupled resin, thereby releasing populations of gene-tagged complexes in their native state. This ‘gene-tagging’ approach has been successfully applied as a C-terminal fusion to the abundant scaffold protein, PSD95 [26], and as an N-terminal fusion (downstream of the signal peptide) on the first extracellular domain of the membrane spanning GluN1 subunit of the NMDAR [5••]. Next, we will describe how these tagging approaches were used to define complexes and supercomplexes containing NMDAR and PSD95.

## Supramolecular organization of the NMDA receptor

The heterotetrameric structure of recombinant NMDAR has been shown in beautiful atomic detail by X-ray crystallography and single-particle cryo-electron microscopy (cryo-EM). These have provided clues to the mechanism of ligand gating [27••,28••,^29^,^30^], albeit with many ‘stabilizing’ mutations and the absence of the entire C-terminal domain (CTD), which accounts for ~1/3 of the protein coding sequence. A recent report revealed that *in vivo* receptors were partitioned into two discrete populations: ~0.8 MDa NMDAR complexes and ~1.5 MDa NMDAR supercomplexes [5••]. The most abundant constituents of the NMDAR supercomplexes in the forebrain were the NMDAR channel subunits (GluN1, GluN2A, and GluN2B) and two Membrane Associated Guanylate Kinase (MAGUK) proteins (PSD95 and PSD93). Combining gene-tagging (as described above) with biochemical methods, these distinct populations of the NMDAR were purified and analysed. NMDAR complexes are composed solely of ion channel subunits, whereas NMDAR supercomplexes contain receptors bound to 50 different proteins including other ion channels, receptors, adhesion proteins, and signalling enzymes [5••].

What molecular mechanisms dictate the assembly of diverse populations of NMDAR supercomplexes? Earlier studies used the cytoplasmic domain of NMDAR GluN2 subunits to ‘fish’ for direct binding partners and this retrieved the four paralogs in the MAGUK family (PSD95, PSD93, SAP102, SAP97), which contain two PDZ domains each and can bind to a short peptide sequence (ES[D/E]V) on the C-terminus of all four GluN2 paralogs (GluN2A-D). This promiscuous *in vitro* interaction between paralogs in two gene families generates combinatorial complexity and predicts that *in vivo* there are potentially sixteen pair-wise interactions that would generate at least as many different supercomplexes. However, rather than the predicted promiscuity found *in vitro*, *in vivo* genetics and biochemistry showed with exquisite selectivity that knockout of either PSD95 or PSD93, blocks the assembly of almost all ~1.5 MDa NMDAR-MAGUK supercomplexes [5••]. Therefore, a single MAGUK protein alone is not sufficient for supercomplex formation; instead both PSD95 and PSD93 are required. Even more striking was the discovery using triple knockin mouse mutations that the canonical PDZ ligand is entirely dispensable for assembling NMDAR-MAGUK supercomplexes. Thus, the binary interactions identified *in vitro* [31,32] have no bearing on specifying the assembly of these proteins at the synapse *in vivo*.

**Figure 3:**
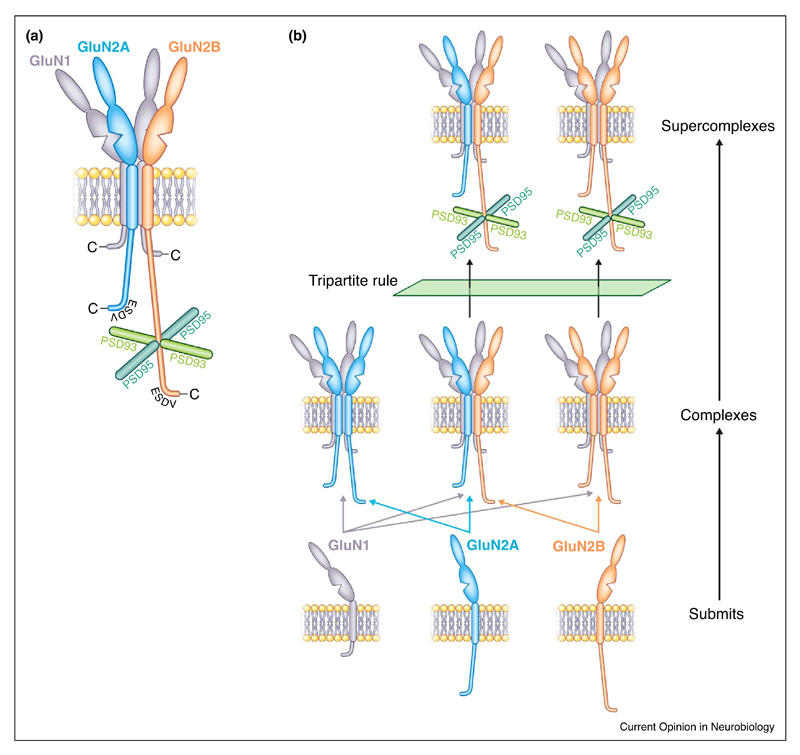
The tripartite rule governs NMDAR-MAGUK supercomplex assembly. **(a)** The tripartite rule describes the genetic requirement of three proteins that are essential for the assembly of NMDA-MAGUK synaptic supercomplexes *in vivo.* Schematic of NMDA receptor subunits (GluN1, GluN2, GluN3) in membrane showing the cytoplasmic tail of GluN2B interacts with PSD95 and PSD93. The assembly of NMDAR-MAGUK supercomplexes does not depend on the ESDV C-terminal PDZ binding site. **(b)** Schematic showing how subunits of NMDA receptors assemble into three receptor complex subtypes and that only those that contain GluN2B can assemble into supercomplexes because of the tripartite rule.

To understand how these two MAGUK proteins interacted with the NMDAR, mice carrying targeted genetic modifications of the cytoplasmic domains of GluN2A and GluN2B were used to identify that the ~600 residue CTD of GluN2B domain was essential, whereas the same domain of GluN2A was not sufficient to mediate supercomplex assembly. Together these findings led to the discovery of the ‘tripartite rule’—PSD95, PSD93 and the GluN2B subunit specify the assembly of NMDAR-MAGUK supercomplexes (Figure 3). In contrast to the earlier models that relied on redundant binary interactions, the tripartite genetic rule provides an example of molecular diversity involving two paralog gene families (Glun2 and Dlg) acting as a molecular gatekeeper to limit the inherent combinatorial diversity of higher-order assembly in the synapse. Similar molecular interdependencies have been found outside of the synapse in unrelated molecular machines composed of multiple paralogous subunits [33]. Thus, the evolution of complex proteomes following two ancestral genome duplications appears to have adopted common mechanisms for specifying self-assembly [34].

## Taxonomy of NMDAR and PSD95 supercomplexes

These genetic studies of the NMDAR family not only reveal that there are two major subfamilies, but it is also apparent that each of these subfamilies can be further divided into many additional members. For example, in the case of the ~0.8 MDa NMDAR complexes, which comprise receptor tetramers, it is a family with three members: di-heterotramers containing GluN2A-GluN1 or GluN2B-GluN1, and GluN2A-GluN2B-GluN1 triheterotetramers (Figure 3b). Although there are only GluN2B-containing di-hetetramers and tri-heterotetramers in ~1.5 MDa supercomplexes, this family is potentially much larger because NMDAR supercomplexes form a population containing various combinations of 50 different proteins.

**Figure 4:**
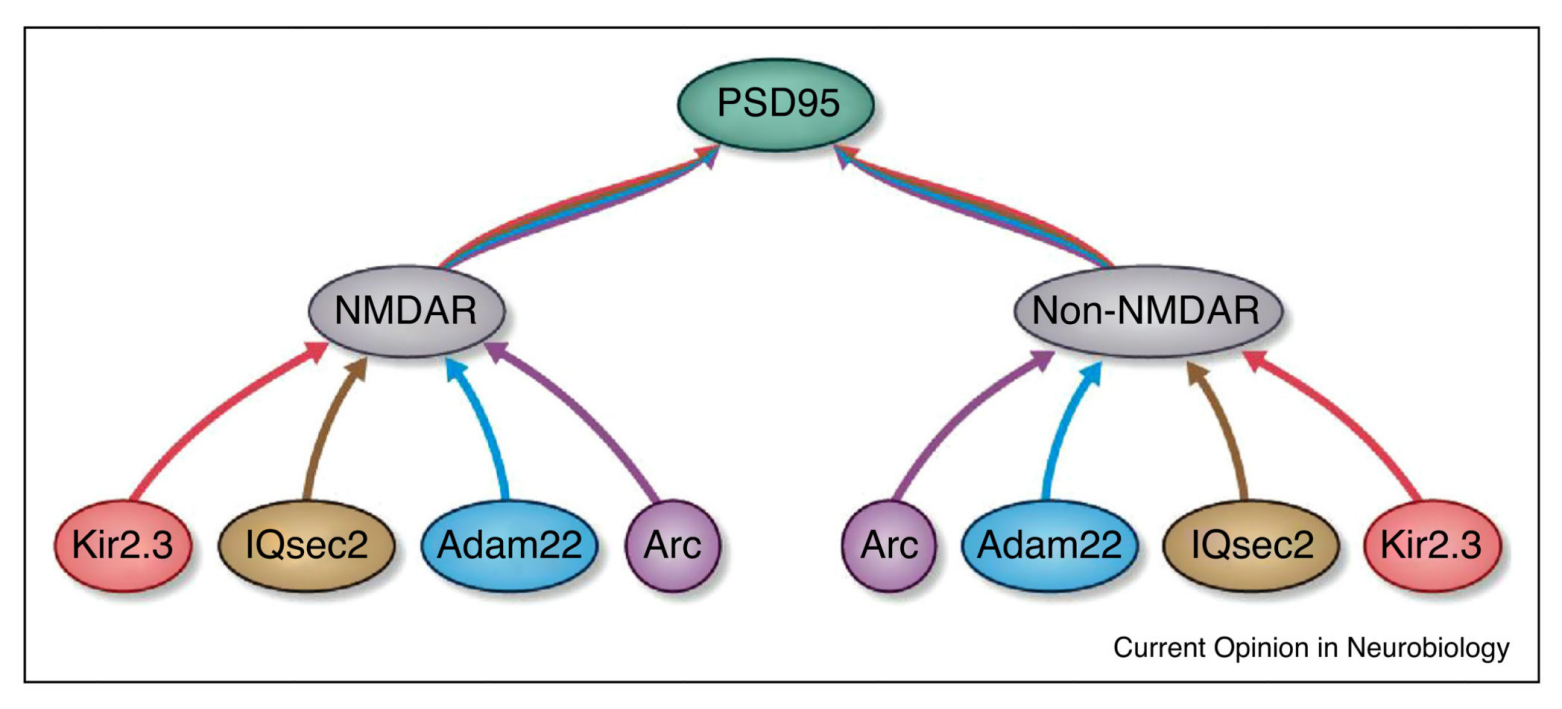
Organization of a family of ~1.5 MDa synaptic supercomplexes. PSD95-containing supercomplexes can be subdivided into a population containing NMDA receptors (NMDAR) and those lacking NMDA receptors (Non-NMDAR). Each of these can be further subdivided into subpopulations according to their assembly with Kir2.3, IQsec2, Adam22 or Arc.

To begin dissecting synaptic supercomplex subfamilies a recent report extended the integrated gene-tagging and biochemical approach to show that in mouse forebrain almost all PSD95 was assembled into ~1.5 MDa supercomplexes, but that only ~3% of these contained NMDARs. Thus, NMDAR supercomplexes represent a subset of a much larger family of PSD95 supercomplexes that do not contain NMDARs (Figure 4). Examining four representative constituents of NMDAR and PSD95 supercomplexes including two membrane-spanning proteins (potassium channel Kir2.3 and adhesion protein Adam22) and two intracellular proteins (a signalling enzyme, IQsec2; immediate-early gene Arc/Arg3.1) showed that these four proteins were in both NMDAR-containing and -lacking supercomplexes. Thus, there are at least eight subfamilies of ~1.5 MDa supercomplexes (Figure 4).

The question then becomes: Are there genetic rules that act as gatekeepers for the assembly of some of these additional proteins? Evidence for some of these supercomplex subtypes has been garnered using mutant mice. For example, Arc and Kir2.3 assembly into ~1.5 MDa supercomplexes required PSD95 but not PSD93 [35•]. Thus, combinations of genetic requirements are likely to represent a general mechanism for specifying the type and composition of synaptic supercomplexes.

Although it will be challenging to test mechanisms of assembly of all synaptic proteins *in vivo*, other evidence suggests the interdependency of supramolecular assembly conferred by genetic rules could be prevalent in axonal complexes [36,37] and the presynaptic terminal [38,39]. An emergent property of combining different genetic requirements for assembly is that greater or lesser selectivity can be controlled by how many constituents of a supercomplex are indispensable. In keeping with this mechanism, multivalent, interdependent assembly has been well characterized in other related non-neuronal MAGUK complexes [40].

Because the essential features of these mechanisms of assembly are that they are genetic and hierarchical, supercomplexes can be specified within a program of development that enables the elaboration of complex synapse proteomes tailored to specific neuronal subtypes and at specific times. Indeed, in mice NMDARs are expressed from neonatal ages but NMDAR supercomplexes are only permitted to assemble after the second postnatal week [5••], consistent with the tripartite mechanism of assembly. The quantity of NMDAR supercomplexes plateaus by the third postnatal week, whereas the population of PSD95 supercomplexes continues to grow into adulthood, consistent with the genetic evidence that supercomplex subfamilies are regulated differentially. Additionally, several other synaptic proteins, including mGluR1/5 and β-catenin, have been found to partition between different supercomplexes in the first and fifth postnatal weeks, respectively [5••]. Thus, the genetic program that determines the timing of gene expression together with the genetic rules for supramolecular assembly control the development of the synapse.

## Postsynaptic nanoclusters

New insight into synaptic architecture at length scales above supercomplexes is being revealed by super-resolution microscopy. Until recently, light microscopy using fluorescent labels could only resolve synaptic proteins as single point spread functions, each marking one synapse from another. With the higher resolution attainable by super-resolution methods, a sub-synaptic architecture of proteins concentrated into nanodomains has been discovered [41–43]. This approach has shown that the apparent concentration of PSD95 varies giving rise to nanodomains (~80 nm diameter) [41,44•]. We estimate this synaptic substructure could accommodate 30–60 supercomplexes (assuming each ~1.5 MDa supercomplex has a diameter parallel to the membrane of 100–200 Å). In another example of the power of tagging endogenous genes in the mouse, PSD95 was fused with enhanced Green Fluorescent Protein (eGFP) or the photoconvertible fluorescent protein probe, mEOS2, and brain sections were imaged using gated Stimulated Emission Depletion (g-STED) microscopy and PhotoActivated Localisation Microscopy (PALM), respectively [44•]. The nanostructure of >100,000 synapses in the circuitry of the hippocampus was examined [44•] and it was clear that there is an architecture to the organisation of synapses, where synapses in different cells and regions of the hippocampus express different numbers of nanoclusters (Figure 1). Interestingly, these knockin fluorescent tags also revealed a peripheral substructure of less concentrated PSD95 outside of the nanocluster that was less evident using antibodies or over-expressed tags. Another recent study showed that pre- and post-synaptic nanodomains may be aligned and thereby positioning the release of neurotransmitter vesicles for optimal activation of postsynaptic supercomplexes [45••]. Although at present it is unclear if genetic rules govern the nanocluster organisation of the synapse, there is evidence that mutations in PSD95 and PSD93 can cause reorganization of the domains within the postsynaptic terminal at the electron microscopic level [46] and biochemically separable compartments [47]. Thus, some of the same genetic rules that are gatekeepers for supercomplex assembly may also play a role at the level of nanodomains.

Nanodomains may also be organized as liquid–liquid phase transition of synaptic constituents. Phase transitions are self-forming protein-rich ‘droplets’ that have been reconstituted *in vitro* using purified, fragments of PSD95 and SynGAP [48••]. These exciting phenomena raise challenging questions, most importantly, to what extent, if at all, these liquid–liquid phase transitions arise *in vivo* with the full-length proteins at physiological concentrations and with other competing interactions that could occlude phase separation. The key requirements for a phase transition, at least *in vitro*, are open, multivalent protein–protein interactions and a very high (sub-millimolar) protein concentration [49]. Other proteins and posttranslational modifications could either facilitate phase transitions by raising the local concentration or abrogate by trapping PSD95 in closed interactions. The assembly of NMDAR-PSD95 supercomplexes is critically dependent on multivalent interactions because in the absence of PSD95 or PSD93, NMDAR supercomplexes fail to assemble. Thus, it will be interesting to understand if supercomplexes compete or complement the phase separated state and reconcile the varying nanodomain architectures of PSD95 detected by super-resolution microscopy [44•].

## Anatomical diversity of synapses

There are two aspects of synapse complexity. First, it is clear that a single synapse contains possibly hundreds of different proteins assembled within multiple molecular machines regulating all aspects of synaptic function. The second factor compounding this complexity is the observation that synapses across different brain regions, neuronal populations, even within the same cell, are compositionally and functionally distinct. Brain region and neuronal-subtype specific expression of synaptic proteins highlights this diversity. The anatomically restricted expression of receptor subunits [50], auxiliary subunits [51] and other constituents [52] indicate synapse composition is highly diverse. A similar pattern of diversity is emerging at the level of supercomplexes. For example, a population of synapses with ~1.5 MDa NMDAR-Kir2.3 ion channel–channel supercomplexes were found enriched in the ventral midbrain, whereas ~1.5 MDa Kir2.3 supercomplexes that lack NMDARs were found in the dorsal cortex [35]. It is likely many other genetic requirements furnish other synapse types with supercomplexes. Indeed, transsynaptic supercomplexes show a similar pattern of anatomical specialization (reviewed elsewhere [53]). As noted above, the PSD95 nanodomain is also anatomically specified, with the CA3 thorny excrescence synapses showing 3–8 nanodomains in contrast to CA1 stratum radiatum that typically contain 1–2 [46]. The capacity to survey large numbers of synapses with super-resolution microscopy opens the door to large-scale brain mapping thereby spanning the nanometre to millimetre length scale. We speculate that the mapping of different complexes, supercomplexes, and nanodomains across the entire neuroanatomy of the brain may reveal spatial patterning that is influenced by the genetic rules of supramolecular assembly.

## Quantifying synaptic composition

Central to understanding the supramolecular organization of synapses is the need to quantify proteins. Quantification of synaptic proteins is important because the relative abundance of subunits necessary for particular molecular machines will contribute to shaping synaptic composition. Mass spectrometric approaches have been useful in estimating the relative and absolute abundance of proteins in the brain [54]. However, these assays usually require upstream biochemical fractionation before quantification and suitable peptide standards. For example, quantifying the molar ratio of GluN2A versus GluN2B by mass spectrometric approaches has not been possible because of the failure GluN2B peptide to ionize [54].

Recently, knockin mouse mutations have served as novel tools to measure the molar ratio of endogenous mouse brain proteins by ‘epitope matching’ the genes encoding different proteins. This approach revealed that GluN2B is four-fold more abundant that GluN2A in the adult forebrain [5••]. This was highly surprising since it had long been assumed that GluN2A was the most abundant GluN2 subunit in adult. Epitope matching using gene-tags has also been used to quantify the relative abundance of synaptic proteins, revealing PSD95 is 17-fold more abundant than GluN1 and that on average each supercomplex contains on average a dimer of PSD95 [35•]. Epitope matching using recombinant chimeric constructs as standards has also been successfully applied to compare the relative abundance of native kainic acid receptor subunits [55].

## Conclusions and perspectives

Over the last twenty years it has become clear that synapses have a remarkable molecular complexity and it is now also evident that this complexity is highly regulated and organized. The supramolecular hierarchy of complexes, supercomplexes and nanodomains represent a framework around which it is possible to describe the architecture of synapses from the atomic to micron length scales. In addition, evidence is now emerging that synapse diversity is the product of the differential distribution of these supramolecular building blocks. It is therefore likely that many of the molecular machines that define each synapse, cell or brain region have yet to be characterized. This framework is also far from complete and one area that will be of great importance is the determination of the substructure of synapses using cryo-electron microscopy and other structural approaches. In the same way that mouse genetic approaches have revealed key principles of synapse organisation and function *in vivo* when combined with biochemical and light-microscopy methods, the versatility of these mouse genetic reagents could, in principle, be applied to study the structure of the synapse by multiple imaging modalities.
